# Relationship Between Sleep-Related Worry, Work Well-Being, and Retention Intention Among Nurses: A Cross-Sectional Multicenter Study

**DOI:** 10.1155/jonm/8839164

**Published:** 2025-09-26

**Authors:** Yanjia Li, Rong Zhang, Limei Kang, Jinlin Li, Zongwen Yi, Haoyue Zhao, Yanyun Su, Yuhang Wu, Feng Chen, Shurong Tang

**Affiliations:** ^1^Department of Emergency, Sichuan Taikang Hospital, Chengdu 610213, Sichuan, China; ^2^Department of Nursing, Sichuan Taikang Hospital, Chengdu 610213, Sichuan, China; ^3^Department of Nursing, Mianyang 404 Hospital, Mianyang 621050, Sichuan, China; ^4^Department of Pediatrics, Sichuan Taikang Hospital, Chengdu 610213, Sichuan, China; ^5^Gynecology and Obstetrics, Sichuan Taikang Hospital, Chengdu 610213, Sichuan, China; ^6^School of Nursing, Meishan Pharmaceutical College, Meishan 620200, Sichuan, China

## Abstract

**Background:** Nurses experience prolonged periods of heavy work pressure, and issues such as poor sleep quality frequently result in work-related anxiety and physical fatigue. Consequently, the academic community has increasingly focused on nurses' work well-being and retention intention. However, the specific impact of sleep-related worry on nurses' work well-being and retention intention remains underexplored.

**Aims:** The purpose of this study was to investigate the relationship between sleep-related worry, work well-being, and retention intention among nurses.

**Methods:** This study adopted a multicenter cross-sectional design and selected 1831 nurses from different medical institutions in China as the research subjects. Data were collected using a self-report questionnaire, including demographic characteristics, the Chinese Nurse Intention to Remain Employed Questionnaire, the Work Well-being Scale, and the Anxiety and Preoccupation about Sleep Questionnaire. IBM SPSS 27.0 was used for descriptive statistics, correlation analysis, single-factor analysis, and mediation effect analysis (Model 4 in PROCESS).

**Results:** The sleep-related worry was significantly negatively correlated with work well-being and retention intention (*r* = −0.434, *p* < 0.01 and *r* = −0.311, *p* < 0.01), the work well-being and retention intention were positively correlated (*r* = 0.576, *p* < 0.01). The work well-being played a mediating role between sleep-related worry and retention intention, with the intermediate effect accounting for 75.86% of the total effect.

**Conclusion:** This study shows that nurses' sleep-related worry not only directly affect their work well-being but also further affect their retention intention through affecting work well-being.

**Implications for Nursing Management:** By investigating the relationship between nurses' sleep-related worry, work well-being, and their retention intention, this study recommends that nursing managers focus on improving nurses' sleep quality, mitigating their sleep-related worry, and offering enhanced support. These measures can effectively increase work well-being, which in turn may strengthen nurses' retention intention and mitigate brain drain.

## 1. Introduction

With the rapid advancement of the global medical industry, the demand for healthcare services has exhibited a pronounced upward trend. In this context, the stability and retention intention of the nursing workforce, as a cornerstone of the healthcare system, have emerged as critical factors influencing the quality of medical services and patient safety [1]. Enhancing nurses' retention intention (i.e., their inclination to continue working in their current roles) is strategically vital for ensuring the stable functioning of the healthcare system [2]. Nevertheless, the ongoing rise in nursing staff turnover has developed into a global public health challenge, drawing significant attention from both academic and professional communities [3]. This trend is driven by multifaceted factors, including the surge in care requirements due to population aging, the diminished social appeal of the nursing profession, and the heightened work pressure resulting from the COVID-19 pandemic and its associated global economic repercussions [4, 5]. It is worth noting that in recent years, the nursing community in China has generally been facing the dual predicament of salary reduction and economic fluctuations [6]. In recent years, China has begun to implement the Diagnosis-Related Groups (DRG) payment reform, promoting the refined operation of medical institutions through cost control and standardized payment [7]. However, this reform has had a profound impact on the career stability of the nurse group, such as the compression of labor costs and the reduction of nurse positions. Most hospitals in China have cut costs by reducing the number of nurses or recruiting more low-paid contract nurses, resulting in the risk of layoffs for regular nurses and frequent resignations for contract nurses due to insufficient salary and benefits [8]. In addition, the workload of nurses has increased and job burnout has intensified. In addition, the DRG payment requirements have shortened the average length of hospital stay and accelerated bed turnover [9]. Nurses need to complete more patient care tasks in a shorter time, resulting in a significant increase in workload. Long-term overwork has led to a decrease in the retention intention. The imbalance between the performance distribution mechanism and salary incentives is another significant impact of DRG payment [10]. After the DRG reform, some hospitals simply transferred the cost control pressure to departments and medical staff, adopting a performance distribution model of “overspending penalties and surplus rewards.” Nurses face income uncertainty because their performance is linked to cost control. Furthermore, the technical charges for nursing services have been persistently low, making it difficult for nurses to obtain reasonable returns through professional contributions. This further weakens their sense of professional achievement and leads to nurse attrition. The high turnover of nurses not only complicates the human capital management of medical institutions but also directly results in the decline in the quality of clinical nursing and the reduction of the operational efficiency of the medical system [11]. Under this realistic background, systematically exploring the current characteristics and related factors of the retention intention of nurses has become an urgent need to optimize the allocation of human resources and ensure the sustainable development of the medical system.

After conducting an extensive literature review, we identified that the existing research on nurses' retention intention primarily focuses on three key areas: personal factors (such as age, gender, and educational background), work environment factors (including interpersonal relationships, work pressure, workload, career development opportunities, compensation, and benefits), and organizational factors (like organizational culture, management style, and leadership approach) [12–14]. For example, Al Sabei et al. [15] revealed that younger nurses might decide to leave their positions due to inadequate career advancement opportunities or difficulties in maintaining work-life balance, while older nurses may prefer to stay because of health concerns or retirement plans. Additionally, Gensimore et al. [16] emphasized that healthcare institutions with positive working environments can more effectively reduce nurse turnover rates and positively impact retention intention. However, there are few reports on the factors related to nurses' positive psychology and the correlation between nurses' sleep-related problems and their retention intention.

Sleep-related worry encompasses an individual's apprehensions about the repercussions of inadequate sleep and diminished control over sleep patterns [17]. This psychological condition is especially common among nurses working in high-stress environments. Given the demands of their profession, nurses frequently encounter shift work, inconsistent rest periods, and extended exposure to high-pressure situations, all of which contribute to poor sleep quality and heightened sleep-related worry [18]. Studies have shown that these sleep concerns can lead to physical and mental fatigue, emotional instability, and decreased work efficiency, ultimately reducing job satisfaction and career commitment [19]. Prolonged sleep deprivation may further intensify job burnout and psychological exhaustion, prompting nurses to view their career prospects less favorably and affecting their retention intention [20]. Moreover, impractical work schedules and elevated stress levels within the workplace can exacerbate nurses' sleep issues, perpetuating a negative cycle [21]. Therefore, it is plausible to propose that sleep-related worry affect nurses' retention intention.

With the rise of positive psychology, work well-being has emerged as a vital component, garnering increasing attention within this field. Work well-being is characterized by employees' self-assessment of their emotional experiences and overall job satisfaction based on personal criteria [22]. This subjective evaluation holds significant importance in the professional lives of nurses. For nurses, work well-being not only their adjustment to and contentment with the work environment but also profoundly impacts their professional identity and long-term career aspirations [23]. Studies have shown a notable negative correlation between work well-being and turnover intentions among nurses [24]. Furthermore, the retention intention of nurses is one of the important factors affecting their turnover rate [25]. Therefore, it can be deduced that there might be a connection between nurses' work well-being and their retention intention.

This study is based on one of the core viewpoints of positive psychology, namely the Conservation of Resources (COR) Theory. This theory emphasizes that individuals cope with stress by obtaining, protecting, and maintaining resources [26]. When resources are lost or threatened to be lost, psychological and behavioral responses will be triggered. Nurses' sleep-related worry can be regarded as a risk of resource loss. Physiological resources (such as sleep quality) are the basis for maintaining an individual's psychological state. Due to the characteristics of shift work and high work intensity, nurses generally have sleep concerns, which directly consume their physiological resources and cause the loss of psychological resources such as emotional exhaustion and cognitive decline, thereby reducing their ability to cope with work pressure [21]. Persistent sleep-related worry may trigger a vicious cycle of “resource loss–increased stress,” further weakening nurses' work well-being. Furthermore, work well-being can also serve as an intermediary mechanism for resource preservation to alleviate resource loss. Nurses with high work well-being are more inclined to make up for the depletion of physiological resources through alternative resources such as positive emotions and social support, thereby maintaining psychological stability, enhancing professional identity, buffering the negative impact brought by sleep concerns, and reducing the risk of job burnout [27]. A positive psychological state can promote the reinvestment of resources and enhance adaptability. The retention intention, as the result of resource reinvestment, means that when individuals perceive that resources are sufficient and can be continuously replenished, they are more inclined to retain the existing resources. For example, nurses will be satisfied with their current professional status and reduce the turnover rate [28]. In addition, there have been many empirical studies in the past that have proved the mediating role of work well-being. The research results of You et al. [29] show that the work well-being of primary nurses has a mediating effect between professional identity and job reshaping ability. Therefore, from a positive psychology standpoint, investigating the interplay between nurses' sleep-related worry, work well-being, and retention intention holds significant value.

In light of the previously discussed context, this research sought to explore the connections among nurses' sleep-related worry, work well-being, and their retention intention, utilizing a multicenter cross-sectional study approach. Furthermore, it investigated whether work well-being serves as a mediator in the relationship between sleep-related worry and retention intention. The findings of this study offer medical institutions a comprehensive, full-chain intervention framework extending from sleep health management to the enhancement of overall well-being. This framework aims to address the issue of high nurse turnover rates. Additionally, it is anticipated to support healthcare organizations in developing evidence-based human resource management strategies while providing theoretical insights and practical recommendations for improving organizational efficiency and employee well-being. Based on theoretical and empirical foundations, this study proposes the following research hypotheses:

  Hypothesis 1: Sleep-related worry is negatively correlated with retention intention.
  Hypothesis 2: Work well-being is positively correlated with retention intention.
  Hypothesis 3: Sleep-related worry is negatively correlated with work well-being.
  Hypothesis 4: Work well-being plays a mediating role between sleep-related worry and retention intention.

## 2. Methods

### 2.1. Study Design, Sample, and Setting

This study was a multicenter cross-sectional correlational study, part of a larger research project to investigate factors associated with hospital nurse retention intentions. The data of this study were collected through online questionnaires from 1831 nurses in 8 hospitals in China (the 8 hospitals included one tertiary hospital in Guangdong Province, one tertiary hospital in Hubei Province, one tertiary hospital in Jiangsu Province, and five tertiary hospitals in Sichuan Province) between January and February 2024 using the convenience sampling method. In the initial phase of the study, participants were selected based on having at least 6 months of clinical experience in general and specialty departments of the selected hospitals. The exclusion criteria were nurses in managerial positions or those working temporarily in hospitals (including interns, etc.). The G-power 3.1 program was used to determine the minimum sample size required for the study with *α* set at 0.05, effect size (*f*^2^) at 0.15, power (1 − *β*) at 0.95, and the number of predictors at 14. It turned out that 129 participants were needed. Considering a 20% attrition rate, this study required at least 154 participants. However, the study initially involved 1840 nurses participating in the questionnaire survey, resulting in 1831 valid responses with a response qualification rate of 99.5%. The actual valid sample size was 1831 cases, far exceeding the theoretical requirements, and the sample robustness was further enhanced through the Bootstrap method (5000 samplings).

### 2.2. Measures

#### 2.2.1. Demographic Characteristics

The questionnaire included sociodemographic characteristics, such as gender, age, marital status, education level, professional title, department, working experience, income, how many night shifts a month, have you experienced a major traumatic event in the past year, and working week.

#### 2.2.2. The Chinese Questionnaire for Nurse Intention to Remain Employed (C-QNIRE)

Nurses' retention intention was measured using the C-QNIRE, which was translated by Tao and Wang [30]. The C-QNIRE consisted of 6 items. The items are rated on a 5-point Likert Scale ranging from 1 to 5. The C-QNIRE ranges from 6 to 30 points. In order to better present the results, the original authors of the C-QNIRE divided their results into three levels based on the total score of the scale: low (6–13), medium (14–23), and high (24–30). The higher the score, the higher the retention intention. Cronbach's alpha coefficient of the C-QNIRE was 0.749 in this study.

#### 2.2.3. The Work Well-Being Scale (WWBS)

The well-being of nurses in their work environment was evaluated using the WWBS, which was developed by Yajing [31]. This scale consists of 15 items, divided into three dimensions: positive emotions (4 items), negative emotions (5 items), and work satisfaction (6 items). Each item is scored on a 5-point Likert scale, with options ranging from 1 (“*totally inconsistent*”) to 5 (“*totally consistent*”). The overall score of the WWBS can range from 15 to 75 points. For easier interpretation of the results, the creators of the WWBS categorized the scores into three levels: low (15–31), moderate (32–58), and high (59–75). In this particular study, the Cronbach's alpha reliability coefficient for the WWBS was 0.929.

#### 2.2.4. The Anxiety and Preoccupation About Sleep Questionnaire (APSQ)

The APSQ was originally developed by Tang and Harvey [32]. This scale was later revised again by Jansson-Frojmark et al. [33]. The Chinese version of APSQ was revised by Shi et al. [34]. It was used to measure sleep-related worries among night-shift nurses. The scale consists of 10 items, and the items are rated on a 5-point Likert Scale ranging from 1 (“*strongly disagree*”) to 5 (“*strongly agree*”). The WWBS ranges from 10 to 50 points. In order to better present the results, the original authors of the APSQ divided their results into three levels based on the total score of the scale: low (10–21), medium (22–38), and high (39–50). The higher scores indicate more severe sleep worries. Cronbach's alpha coefficient of the APSQ was 0.968 in this study.

### 2.3. Data Collection

We utilized convenience sampling to randomly select 8 hospitals in China. With the support of members from a professional academic organization, we distributed questionnaires at each selected hospital. Prior to the survey, we conducted comprehensive training for the research team. Researchers rigorously screened potential participants according to predefined inclusion and exclusion criteria. Subsequently, they informed the participating nurses about the study's objectives and significance, as well as detailed instructions on how to complete the questionnaire and the deadline for submission, via social communication platforms such as WeChat. After obtaining consent from the respondents, the researchers provided them with links to the online questionnaires for data collection. To ensure the accuracy and reliability of the data, two investigators jointly reviewed the completed questionnaires to verify their completeness.

### 2.4. Ethics Statement

This study received approval from the Ethics Review Committee of Sichuan Taikang Hospital (#SCTK-IRB-2024-014). Despite the online survey format, which precluded obtaining written consent, strict adherence to ethical guidelines was maintained. Prior to participation, all respondents were required to confirm their agreement by clicking a checkbox on the survey login page. This checkbox provided detailed information regarding the research objectives and methods, potential risks and benefits of participation, and the participants' right to withdraw from the study at any time without any repercussions.

### 2.5. Data Analysis

Before conducting hypothesis testing, an initial analysis was performed. In this research, IBM SPSS 27.0 was used to carry out normality tests, descriptive statistics, and correlation analyses for all variables under study. An exploratory factor analysis was also conducted to ensure the reliability of the scale. Furthermore, one-way ANOVA and *t*-tests were applied to investigate demographic differences in nurses' retention intention. To evaluate the mediating effect of work well-being on the relationship between sleep-related worry and retention intention, bootstrapping was conducted using Model 4 of Hayes' PROCESS macro in IBM SPSS 27.0. The significance of the mediation effect was assessed using a 95% bias-corrected confidence interval. Statistical significance was set at *p* < 0.05 with two-tailed testing. In addition to the preset mediation model, this study conducted an exploratory analysis using PROCESS Model 1 to examine the potential moderating role of work well-being. The results indicated that the interaction effect was not statistically significant (*p* > 0.05), thereby lending greater support to the mediation model as a more appropriate explanatory framework.

## 3. Results

### 3.1. Personal Characteristics and Single-Factor Analysis of Retention Intention

In this study, 1831 nurses from eight hospitals participated. The sample predominantly comprised female (97.4%, *n* = 1783), and the majority held at least a bachelor's degree (71.4%, *n* = 1308). Additionally, a relatively small proportion of participants were single, divorced, or in other nonmarried statuses (26.4%, *n* = 483). Approximately half of the nurses earned less than 5000 RMB (44%, *n* = 805) monthly. Significant differences were observed in the nurses' retention intention based on gender, age, marital status, professional title, working experience, income, how many night shifts a month, have you experienced a major traumatic event in the past year, and working week (*p* < 0.05) (Table 1).

### 3.2. Descriptive Statistics of Main Study Variables

In this study, the total mean score of nurses' sleep-related worry, work well-being, and retention intention were 30.40 ± 11.28, 53.09 ± 10.8, and 22.79 ± 3.71 (Table 2).

### 3.3. Correlation Among Major Variables

In this study, nurses' sleep-related worry was negatively correlated with the work well-being (*r* = −0.434, *p* < 0.01) and the retention intention (*r* = −0.311, *p* < 0.01). In addition, the work well-being is also positively correlated with the retention intention (*r* = 0.576, *p* < 0.01) (Table 3).

### 3.4. Mediating Effect of Nurse's Work Well-Being, Between Sleep-Related Worry, and Retention Intention

**Figure 1:**
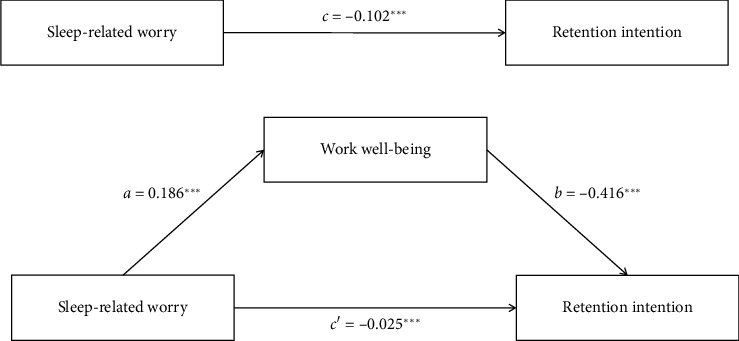
Mediation model of work well-being on the relationship between sleep-related worry and retention intention of nurses. ^∗∗∗^*p* < 0.001.

The study assessed the mediating role of work well-being on the relationship between sleep-related worry and retention intention. The results showed that sleep-related worry significantly predicted work well-being (*a* = 0.186 and SE = 0.007, *p* < 0.001), work well-being was shown to be a significant predictor of medical narrative ability (*b* = −0.416 and SE = 0.020, *p* < 0.001), and sleep-related worry also had a direct effect on retention intention (*c*′ = −0.025 and SE = 0.007, *p* < 0.001). The bias-corrected percentile Bootstrap method test showed that 95% CI did not include 0, indicating that work well-being partially mediated the relationship between sleep-related worry and retention intention. The mediation effect accounted for 75.86% of the total effect (Table 4 and Figure 1).

## 4. Discussion

As the backbone of the medical profession, nurses' sleep-related worry, work well-being, and retention intention are not only related to the quality of care but also affect the sustainable development of the medical system. Recently, there has been a notable rise in mental health challenges, particularly sleep-related issues, among nursing professionals [35]. Such challenges can adversely affect both the physical and psychological well-being of nurses, which may result in lower job satisfaction and higher turnover rates [36]. Therefore, this study seeks to offer a fresh theoretical framework and practical recommendations for enhancing nurse management and boosting retention intention by exploring the interconnections between sleep-related worry, work well-being, and retention intention.

The findings of this study revealed that nurses' retention intention was at a moderate level, aligning with results from previous research [37]. Globally, there is a noticeable decline in nurses' retention intention, particularly in high-stress healthcare environments [38]. This trend is especially evident in China, where factors such as heavy workloads, frequent night shifts, irregular schedules, and job burnout significantly influence nurses' decisions to leave or transfer, leading to lower retention intention rates [39]. In this study, various demographic and professional characteristics were found to affect nurses' intentions to stay. Specifically, older nurses with higher educational attainment, longer tenure, higher incomes, fewer night shifts, no major traumatic events in the past year, and less overtime demonstrated a stronger inclination to remain in their positions. Conversely, nurses with contrasting characteristics showed lower retention intention. Previous studies have also emphasized that low social status, insufficient income, high work intensity, and inadequate organizational support contribute to organizations undervaluing the economic importance of nurses, resulting in higher turnover rates [40]. Therefore, it is recommended that hospital and nursing administrators enhance their support for career development and education [41] (for instance, by implementing a structured academic advancement program, collaborating with universities to offer part-time master's or doctoral programs in nursing, providing tuition subsidies ranging from 50% to 70%, and offering flexible study schedules. Additionally, the establishment of an “expert nurse” title sequence within hospitals could be considered, enabling senior nurses to engage in departmental management and decision-making processes). Second, the incentives for long-term contributors can be strengthened, such as awarding customized MEDALS and an additional 7 days of leave at the 5th, 10th, or 20th year of an individual's employment, and providing a one-time bonus. The longer the tenure, the higher the bonus [42]. Additionally, the salary and welfare structure can be improved and optimized, such as implementing night shift salary reform and providing salary subsidies for night shift nurses. Those who work night shifts no less than four times a month can choose to have a 2-day weekend off on their own or accumulate one day of compensatory leave [43]. A comprehensive income growth mechanism can also be established. A total of 20% of the surplus of drug consumption in the department can be converted into nurse bonuses, and special rewards can be set up for nurses engaged in nursing research and clinical work [44]. Finally, it is crucial to develop mental health services to help nurses manage work-related stress. For instance, hospital administrators should regularly organize mental health knowledge lectures, and department heads should also frequently pay attention to the psychological conditions, family situations, and social environments of nurses. For nurses who have experienced major life events or changes, timely assistance and support should be provided, and appropriate psychological counseling should be offered when necessary [45].

The results of this study show that there is a significant negative correlation between nurses' sleep-related worry and their work well-being. This means that the higher the degree of concern nurses have about sleep problems (such as worrying about poor sleep quality, difficulty falling asleep, or insufficient sleep), the lower the level of work well-being they experience. This negative correlation indicates that sleep-related worry is an important negative factor affecting nurses' work well-being. Specifically, sleep-related worry can lead to low mood and physical and mental fatigue among nurses, constantly depleting their emotional and physical resources. These negative physical and mental states further undermine nurses' work satisfaction and overall work well-being. Therefore, in order to alleviate nurses' sleep-related worry and enhance their work well-being and the quality of care, it is suggested that medical institution managers and individual nurses take the following measures: for instance, organizing mindfulness workshops (offering structured mindfulness courses such as mindful breathing, body scans, and mindful walking), introducing guided meditation resources (providing recommendations for high-quality meditation apps like Headspace and Calm, or internally recorded short meditation audio clips (5–15 min) for nurses to use during commuting, lunch breaks, or before going to bed), providing basic courses in cognitive behavioral therapy (teaching skills for identifying and changing negative thinking patterns, such as catastrophizing thoughts, that cause sleep concerns and work stress), optimizing shift scheduling and providing rest guarantees (managers should establish a more humanized shift scheduling system, such as avoiding frequent day and night shifts, ensuring continuous rest days, and ensuring that nurses have genuine rest time after work hours, being able to leave the work area without being disturbed, and reducing the frequency of training visits to the hospital during night shift rest time) [46, 47].

The results of this study also show that nurses' sleep-related worry is negatively correlated with their retention intention. Studies show that persistent sleep-related worry are often accompanied by actual sleep deprivation or low-quality sleep, which directly leads to physical fatigue, cognitive decline (such as inattention and weakened judgment), and emotional exhaustion (such as irritability, depression, and emotional numbness) [48]. In such a state of physical and mental exhaustion, nurses find it difficult to effectively cope with the high pressure and emotional demands of clinical work. Their perception of workload is significantly magnified, and they are more likely to have the idea of evading (quitting). Second, fatigue and cognitive impairment caused by sleep problems will increase the risk of errors at work and reduce work efficiency. This may not only undermine nurses' sense of professional efficacy and achievement but also cause job insecurity and anxiety due to the fear of making mistakes. Negative work experiences and the lack of a sense of security are important factors that reduce nurses' retention intention in their positions. Therefore, nursing managers should implement a “sleep-friendly” shift system, reduce rapid rotations, avoid frequent switching between day shifts, night shifts, and evening shifts, and adopt longer shift cycles (such as giving sufficient rest after several consecutive night shifts) to allow the biological clock to adapt [49]. On the one hand, night shift arrangements can be optimized: limit the number of consecutive night shifts and avoid arranging early shifts or important meetings immediately after night shifts. On the other hand, sleep health education and skills training can be systematically promoted, such as teaching cognitive behavioral therapy (CBT-I) techniques for dealing with insomnia: for sleep anxiety, train nurses to identify and change catastrophic thinking about sleep, establish a positive connection between the bed and sleep (stimulus control), and create a regular sleep-wake schedule (even on rest days, try to stay as consistent as possible) [50]. The most important thing is that the nursing department or hospital administrators should also train head nurses so they understand the importance of sleep for nurses' health, safety, and retention intention. Encourage managers to proactively and noncritically inquire about nurses' sleep conditions and challenges and show care in shift scheduling and temporary task arrangement.

Nurses with a higher level of work well-being are more likely to have a stronger retention intention. Conversely, a low level of work well-being is an important early warning signal of the intention to resign. This discovery highlights the strategic significance of enhancing work well-being for stabilizing the nursing team. Studies show that high work well-being stems from a profound recognition of the professional value of nursing, job autonomy, and a sense of achievement [51]. When nurses feel the meaning of their work (such as the satisfaction of saving lives) and gain growth opportunities, they will form a strong professional commitment, thereby enhancing their retention intention. Second, nurses with a high sense of happiness usually have more abundant emotional resources (such as positive emotions and psychological resilience) and can effectively cope with work pressure and emotional exhaustion. This psychological protection mechanism reduces the risk of having the idea of quitting due to burnout. Therefore, it is suggested that nursing managers take corresponding measures to improve nurses' work well-being, which is conducive to enhancing their retention intention. For example, they can reshape the sense of job value and provide professional growth support (establishing a career development ladder system, designing a clear promotion channel (clinical expert to management position to teaching position), and providing skill certification and salary increases for each level). Additionally, they can build an emotionally supportive working environment (providing free mindfulness courses, group art therapy, and one-on-one psychological counseling), and effectively manage workload and autonomy (intelligent shift scheduling and flexible management, integrating nurses' preferences, fatigue index, and skill matching degree, ensuring continuous rest of at least 48 h per week, and mandatory rest for at least 24 h after night shifts should be provided). Finally, providing organizational support is also very important (such as parental leave, holiday allowances, night shift subsidies, subsidies for after-school care for children, and discounted services for elderly home care) [52].

One of the key insights from this study is that nurses' work well-being plays a vital mediating role between sleep-related worry and retention intention. This suggests that sleep-related worry not only directly affects retention intention but also exerts an indirect influence through work well-being. The findings highlight the importance of boosting nurses' work well-being to counteract the negative effects of sleep concerns on retention intention. When nurses feel psychologically fulfilled, experience positive emotions, and find value in their work, they are more likely to remain in their roles. On the other hand, nurses who experience low energy or mood due to sleep concerns may struggle to derive satisfaction and a sense of accomplishment from their work, which can reduce their retention intention [53]. While directly addressing sleep concerns might have limited effectiveness, enhancing nurses' work well-being can significantly mitigate the adverse impact of sleep-related worry on retention intention [54]. Furthermore, the COR Theory also provides a powerful explanatory framework for understanding the relationship among nurses' sleep-related worry, work well-being, and retention intention [26]. The mediating role of work well-being is essentially a dynamic manifestation of the resource stock depletion process, while the decline in retention intention is the ultimate manifestation of resource defense behavior. The essence of this mediating effect is a chain depletion process of resources. Sleep is a key way for the recovery of physiological resources in the human body. However, sleep-related worry continuously consumes physiological resources (insufficient physical recovery) and emotional resources (increased anxiety), thereby gradually leading to the collapse of retention intention, reduced professional identity and work efficiency, and even adverse nursing events or errors, causing nurses to have self-doubt. This affects team collaboration and work quality, reduces their sense of belonging to the organization, and ultimately influences their retention intention. Moreover, when resource depletion causes work well-being to drop below the threshold and nurses perceive an imbalance between resource input and return, the “resource protection motivation” will be triggered, and they will regard resignation as a survival strategy. For example, if they leave the high-pressure environment to avoid physical and mental breakdown, their retention intention will also decrease significantly. Therefore, in order to break this vicious circle and reduce the loss of nursing talents, hospitals and nursing managers should actively take corresponding measures to enhance nurses' retention intention. For example, supplementary physiological resources can be adopted (such as scientifically scheduling shifts to ensure sleep opportunities and adopting a “fixed shift cycle system,” the same nurse should be in the same type of shift for four consecutive weeks to avoid frequent switching between day and night). Second, emotional resources can be reconstructed (such as through mindfulness training to enhance emotional resilience, conducted once every quarter), and a teasing session can also be held once a month, which is beneficial for nurses to vent their emotions. Additionally, cognitive resources can be enhanced (such as skills training to reduce the cognitive load of work). In addition, social resources can be strengthened in several ways (such as team support to enhance a sense of belonging, setting up an “emotional first aid baton”: when a nurse turns on an emotional yellow light, a partner can take over their nonurgent tasks for 20 min, and establishing a “Nursing Family Support Association”: providing free care for children of nurses working night shifts until 10 p.m. and offering a green channel for emergency medical accompaniment for elderly family members) [55, 56].

## 5. Advantages and Limitations

This study constructed for the first time a theoretical model in which sleep-related worry affects nurses' retention intention through work well-being, revealing the chain mechanism of physiological, psychological, and occupational behaviors. It provides interdisciplinary theoretical support and practical paths for nursing managers, as well as a new intervention direction for medical institutions—enhancing nurses' work well-being by improving their sleep-related worry so as to strengthen their retention intention rather than relying solely on organizational management measures. Second, most existing studies focus on the influence of the working environment or psychological capital on nurses' retention intention, but few studies incorporate sleep-related worry as an independent variable into the analytical framework. This study takes “sleep-related worry,” a comprehensive physiological–psychological factor, as an independent variable for the first time, revealing its influence path on the willingness to stay and expanding the dimension of research on nurses' professional behaviors. More importantly, this study also features interdisciplinary innovation: combining a multidisciplinary perspective of medicine (sleep-related worry), psychology (work well-being), and management (retention intention), it breaks through the limitation of traditional nursing research that focuses on a single field.

Despite its valuable contributions, the study has certain limitations. First, the cross-sectional design fails to reveal the temporal relationships among variables, which may lead to misjudgment of the mediating effect and thereby weaken the causal explanatory power of the research conclusion. Therefore, it is suggested that subsequent studies can innovate research methods, adopt longitudinal research designs, and combine intervention experiment designs to verify the hypothesized causal paths at the same time. Second, the homologous bias of self-reported data may amplify the pseudocorrelation among variables. Future research suggests conducting data calibration by integrating multimodal data through triangulation verification methods, and qualitative interviews can also be used to further verify the research results. Third, samples with a single cultural background may limit the ecological validity of the model. The hierarchical medical treatment system unique to the Chinese medical system and cultural psychological factors may affect the association patterns among variables. Therefore, cross-cultural research is necessary. It is suggested that the subsequent research construct a cross-cultural collaboration network and use the equivalence test to compare the measurement invariance of samples from China and the West. Although the theoretical and data results of this study focus on the mediating role of work well-being, future research can further explore its dynamic moderating mechanism in sleep-related worry and retention intention, and the complex path relationship can also be further verified through SEM or longitudinal design. Finally, uncontrolled confounding variables may lead to bias due to omitted variables. Future research is suggested to consider, in addition to the covariates that have been accounted for, other biological or environment-related variables as well. Machine learning algorithms could also be employed to automatically screen for confounding factors, thereby enhancing the credibility of the research results.

## 6. Conclusions

In this multicenter cross-sectional study, we examined how nurses' work well-being mediates the relationship between sleep-related worry and retention intention. Our findings indicate that sleep-related worry indirectly affects nurses' retention intention through their level of work well-being. This underscores the critical need to address sleep-related issues and enhance job contentment to support nurses' career development. The study emphasizes that healthcare administrators should adopt holistic strategies to reduce nurses' sleep-related worry and increase their work well-being, thereby fostering a more supportive working environment. Such initiatives can boost nurses' commitment to their roles, offering valuable insights and evidence for future human resource management and occupational health interventions. Ultimately, these efforts contribute to the sustainable advancement of the healthcare system.

## 7. Implications for Nursing Management

This study reveals the complex mechanism of how nurses' sleep-related worry impacts their retention intention through work well-being and also reveals the key role of work well-being, which provides important practical guidance for nursing management and policymakers. This can be accomplished by addressing nurses' sleep health and enhancing their work well-being. Therefore, it is suggested that nursing managers should arrange shifts reasonably (avoid frequent shift changes and ensure at least 48 h of rest time after night shifts), conduct sleep repair workshops such as implementing CBT-I insomnia treatment (group training twice a month), and improve the working environment (equip the duty room with light-blocking eye masks and soundproof earplugs, etc.). Such initiatives can help counteract the negative impact of sleep-related worry on nurses' retention intention. More importantly, multilevel career development incentives should be provided (for example, by establishing promotion channels for clinical experts, management positions, and teaching positions. For each promotion by one level, the salary increase should be no less than 12%). By deepening cooperation with educational institutions to enhance the professional educational level of nurses, improving the psychological support system (such as holding a mental health lecture once every quarter), and creating a harmonious working atmosphere and team culture (such as organizing regular team-building activities), the work well-being of nurses can be comprehensively enhanced. These efforts will enhance nurses' sense of professional value, leading to greater work well-being and higher retention intention. Ultimately, this approach will not only help mitigate the nursing shortage but also significantly enhance patient care quality and overall healthcare system efficiency.

## Figures and Tables

**Table 1:** Personal characteristics and single-factor analysis of retention intention (*N* = 1831).

Variables	*N* (%)	*M* ± SD	*t*/*F*	*p*
Gender			2.923	0.004
Female	1783 (97.4)	22.83 ± 3.68		
Male	48 (2.6)	21.25 ± 4.38		
Age (years)			17.888	< 0.001
18–25	210 (11.5)	21.70 ± 3.60		
26–30	601 (32.8)	22.33 ± 3.79		
31–35	500 (27.3)	22.71 ± 3.56		
36–40	259 (14.1)	23.21 ± 3.59		
41–45	124 (6.8)	24.18 ± 3.32		
46–60	137 (7.5)	24.72 ± 3.48		
Marital status			19.000	< 0.001
Married	1348 (73.6)	23.06 ± 3.70		
Single	410 (22.4)	21.81 ± 3.60		
Divorce or others	73 (4.0)	23.37 ± 3.45		
Education level			−0.465	0.642
College	523 (28.6)	22.73 ± 3.71		
University or above	1308 (71.4)	22.82 ± 3.71		
Professional title			14.05	< 0.001
Nurse	267 (14.6)	22.21 ± 3.63		
Senior nurse	738 (40.3)	22.41 ± 3.67		
Nurse-in-charge	701 (38.3)	23.15 ± 3.72		
Associate chief nurse or above	125 (6.8)	24.28 ± 3.48		
Department			1.808	0.094
Internal medicine	688 (37.6)	22.54 ± 3.80		
Surgical	432 (23.6)	22.75 ± 3.71		
Pediatric	86 (4.7)	23.34 ± 3.12		
Obstetrics and gynecology	115 (6.3)	23.33 ± 3.19		
Outpatient	83 (4.5)	23.43 ± 3.79		
Emergency	87 (4.8)	22.55 ± 3.72		
Others	340 (18.6)	22.93 ± 3.76		
Working experience (years)			19.510	< 0.001
3	212 (11.6)	21.77 ± 3.66		
3–5	319 (17.4)	22.38 ± 3.67		
6–10	426 (23.3)	22.23 ± 3.75		
11–15	521 (28.5)	23.04 ± 3.62		
> 15	353 (19.3)	24.08 ± 3.47		
Income (RMB/month)			10.083	< 0.001
3000	106 (5.8)	21.32 ± 4.01		
3000–5000	699 (38.2)	22.64 ± 3.66		
5001–10000	978 (53.4)	22.98 ± 3.69		
10,000	48 (2.6)	24.44 ± 2.87		
How many night shifts a month			17.287	< 0.001
0	406 (22.2)	23.66 ± 3.63		
1–4	569 (31.1)	23.02 ± 3.58		
5–8	455 (24.8)	22.51 ± 3.66		
8	401 (21.9)	21.90 ± 3.79		
Have you experienced a major traumatic event in the past year			2.728	0.006
No	1703 (93.0)	22.85 ± 3.70		
Yes	128 (7.0)	21.93 ± 3.78		
Working week (hours)			14.568	< 0.001
40	195 (10.6)	23.59 ± 3.32		
40–45	1038 (56.7)	23.03 ± 3.60		
46–50	402 (22.0)	22.43 ± 3.94		
50	196 (10.7)	21.46 ± 3.75		

**Table 2:** Descriptive analyses of main study variables (*N* = 1831).

Variables	Min	Max	*M* ± SD
Sleep-related worry			
Total score	10	50	30.40 ± 11.28
Work well-being			
Positive emotion	4	20	13.93 ± 3.55
Negative emotion	5	25	18.19 ± 5.36
Work satisfaction	6	30	20.97 ± 4.84
Total score	15	75	53.09 ± 10.81
Retention intention			
Total score	6	30	22.79 ± 3.71

**Table 3:** Correlation analyses between sleep-related worry, work well-being, and retention intention (*N* = 1831).

Variables	Sleep-related worry	Work well-being	Retention intention
Sleep-related worry	1		
Work well-being	−0.434^∗∗^	1	
Retention intention	−0.311^∗∗^	0.576^∗∗^	1

^∗∗^ *p* < 0.01.

**Table 4:** Mediating effect of work well-being between sleep-related worry and retention intention (*N* = 1831).

Effect	Path	*β*	Bootstrap 95% CI	SE	*t*	*p*
Direct effect	Sleep-related worry ⟶ Retention intention	−0.025 (*c*′)	−0.038, −0.011	0.007	−3.555	< 0.001

Indirect effect	Sleep-related worry ⟶ Work well-being	0.186 (*a*)	0.172, 0.201	0.007	25.704	< 0.001
	Work well-being ⟶ Retention intention	−0.416 (*b*)	−0.455, −0.376	0.020	−20.600	< 0.001

Total effect	Sleep-related worry ⟶ Retention intention	−0.102 (*c*)	−0.117, −0.088	0.007	−13.997	< 0.001

*Note:* Adjusting for covariates, including gender, age, marital status, professional title, working experience, income, how many night shifts a month, have you experienced a major traumatic event in the past year, and working week.

Abbreviations: CI = Confidence interval, SE = standard error.

## Data Availability

The data that support the findings of this study are available from the corresponding author upon reasonable request.
